# Supplementary figures and images for: Tympanoplasty and adenoidectomy in children: Comparison of simultaneous and sequential approaches
Source: PLoS One. 2022 Mar 10;17(3):e0265133. doi: 10.1371/journal.pone.0265133 (PMC8912196; doi:10.1371/journal.pone.0265133)

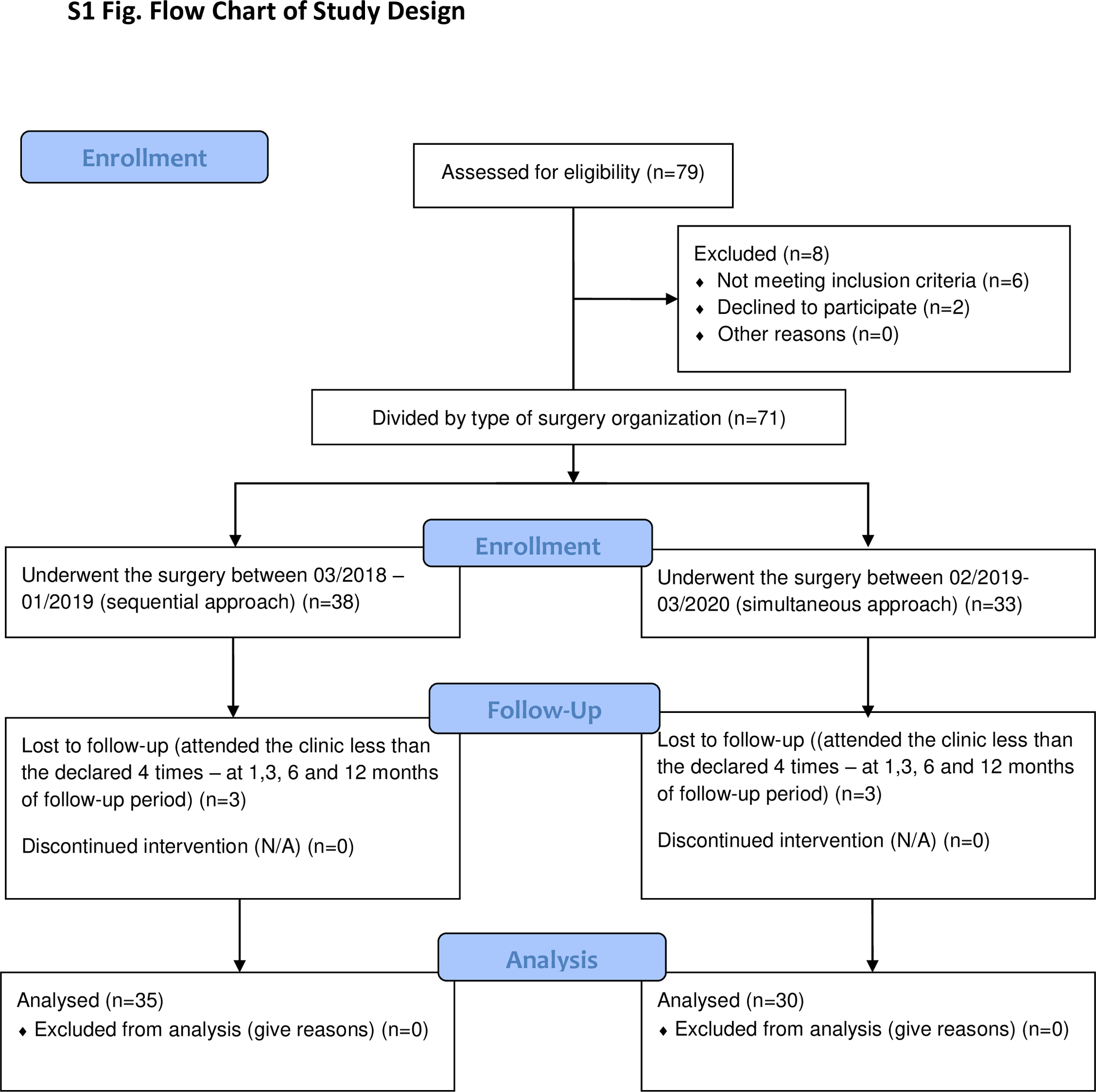

Supplement: S1 Fig — (TIF) [file pone.0265133.s001.tif]
