# Supplementary material for: Tympanoplasty and adenoidectomy in children: Comparison of simultaneous and sequential approaches
Source: PLoS One. 2022 Mar 10;17(3):e0265133. doi: 10.1371/journal.pone.0265133 (PMC8912196; doi:10.1371/journal.pone.0265133)
Supplement: Attachment — Submitted filename: Response to Reviewers.docx [file pone.0265133.s003.docx]

# Response to Reviewers:

# Journal: PLOS ONE

**Manuscript No:** PONE-D-21-32269

**Manuscript title:** Tympanoplasty and Adenoidectomy in Children: Comparison of Simultaneous and Sequential Approaches

**Authors:** Shamshudinov et al.

| **Reviewer 1** | **Changes by the authors** |
| --- | --- |
| The topic of the paper is interesting and sound scientifically relevant. However, some major corrections have to be performed to improve its quality before publication. | Thank you very much for reviewing of our manuscript. We will try to give detailed answers to all comments.  We highlighted all changes made in yellow |
| Background:  Line 5: In children, Eustachian Tube is less functional than the adult one and it depends on its different anatomical position. Eustachian tube function has been evaluated with semi-objective method (ETS-7) and objective one (CT) that gives information about its anatomical-functional activity and the relation with COM (atelectasis, OME, and cholesteatoma). cite <https://doi.org/10.1016/j.anorl.2017.11.009>; doi: 10.1371/journal.pone.0247708. | Done. We added the statement using these references.  Notably, Eustachian tube disfunction detected with semi-objective (Eustachian tube score; ETS-7) and objective (computed tomography combined with Valsalva maneuver) methods is associated with high prevalence and incidence of COM in children [4,5]. |
| Methods:  Line 2: You said that the retrospective study was conducted from 03/2018 to 03/2021, but it is in contrast with sentences reported in line 61 (follow up at 12 months) and line 6 of “results” where you define preoperative and postoperative ABG after 12 months. Have you made a mistake? Was it 2020? please clarify this point or define patients that have ended the follow-up and patients that are at about 6 months.  Line 41: How were patients medically treated after surgery? Please add pharmacological protocol performed (es: doi: 10.14639/0392-100X-N0150). | Thank you. We corrected this technical mistake  This retrospective, comparative, non-randomized clinical study was conducted between 03/2018 and ~~03/2021~~ 03/2020 at the Ear Nose Throat department of General Hospital #5 of Almaty City, Kazakhstan.  Thank you. We added the pharmacological treatment to the text of manuscript.  All patients were prescribed a course of oral antibiotics during postoperative 1 week (type of antibiotic was selected according to patients’ sensitivity). Additionally, non-steroidal anti-inflammatory drugs (acetaminophen, ibuprofen, ketoprofen) were given in all children in case of fever or pain. |
| Results:  Line 6: add how many patients performed contemporary tympanoplasty and ossiculoplasty. | Thank you. Done.  The ossiculoplasty was performed in three and four patients of simultaneous and sequential groups respectively. |
| Discussion: Line 32: Currently it has been shown with semi-objective method (Eustachian Tube Score 7) that children with Adenoid Hypertrophy, have a high incidence of Eustachian Tube Dysfunction (cite doi:10.1177/0145561321989455). | Thank you. We added this statement to the text of manuscript.  Currently it has been shown with semi-objective method (ETS-7) that children with adenoid hypertrophy have a high incidence of Eustachian tube dysfunction [23] |
| Table 3: Line of complication add “0” or “/” in the empty spaces. | Thank you. We removed the subheading “Complication” related to the pain rate from table. |
| References: Correct the references (1-3 are reported twice) |  |

| **Reviewer 2** | **Changes by the authors** |
| --- | --- |
| Minor suggestions: | Thank you. Your comments were very valuable to us.  We highlighted all changes made in yellow |
| Background  Line 3, change studies with evidence from literature demonstrates...  line 19, Although a greater number of sequelae is described in the use of the ventilation tube, the correct stratification of the patients allows the most appropriate approach depending on both the clinical and instrumental presentation. The surgical method is affected by the operator's experi- ence. Other factors that infuence prognosis are the age of the patient at the time of initial placement of the tube, concomitant adenoidectomy, the type of ventilation tube, the duration of maintenance in the ventilation tube, regular follow-up by the same otologist and strict water precautions. please cite Ferlito S, Cocuzza S, Grillo C. Complications and sequelae following tympanostomy tube placement in children with effusion otitis media: Single center experience and review of literature. Acta Medica Mediterranea 36(3). doi:10.19193/0393-6384_2020_3_298.  line 29, Myringotomy and tube insertion combined with balloon eustachian tuboplasty has been demonstrated an effective and safe in the treatment of children with OME. Directly benefit from the ventilation tube, the curative effect was close during the period of tube retention. please discuss and cite Chen S, Zhao M, Zheng W, Wei R, Zhang B, Tong B, Qiu J. Myringotomy and tube insertion combined with balloon eustachian tuboplasty for the treatment of otitis media with effusion in children. Eur Arch Otorhinolaryngol. 2020 May;277(5):1281-1287. doi: 10.1007/s00405-020-05828-9. Epub 2020 Jan 30. PMID: 32002612. | Thank you. We paraphrased this sentence.  The evidence from literature demonstrates that COM negatively affects children's and caregivers' well-being and quality of life  Thank you. We added this sentence to the text of manuscript.  The recent study of Ferlito et al. demonstrated that concurrent adenoidectomy was the contributing factor to the lower incidence of post-operative otorrhea in children with OME [12]  Thank you. We added this sentence to the text of manuscript.  Moreover, even the enhancement of Eustachian tube function alone may contribute significantly to the curative process of OME. Balloon eustachian tympanoplasty combined with myringotomy and tube insertion was recognized as efficient intervention in the treatment of OME in children [17] |
| Methods - apply the consort guidelines - perform a flow diagram describing the protocol  - describe clearly selection criteria | Thank you. We tried to adjust consort guidelines for flow diagram of our retrospective, comparative, non-randomized clinical study (please see Supporting information – S1 Table)  Thank you. The inclusion criteria are given in the second paragraph of “Methods” section   - Age 3-17 years - Follow-up period – 12 months - Diagnosis of COM combined with adenoid hypertrophy - The types of intervention – power-assisted adenoidectomy and type 1 tympanoplasty (at the same day or sequentially) |
| Results - clear and well written - always report both percentage and number or value | Thank you. We added numbers to Table 3 |
| Discussion  - Adenotonsillectomy have been reported by several authors as a curative approach in children for both sleep apnea and behavioural disorders. Please discuss and cite doi: 10.1016/j.ijporl.2005.08.008. and doi:10.3390/children8100921.  - Children with down syndrome demonstrated to undergo more repeat tube insertion (tti )if they were of younger age and if the indication for surgery up to 61%. please cite doi:10.1016/j.ijporl.2021.110811 | Thank you. We added this data to the text of manuscript.  The existing data demonstrated the curative effect of adenotonsillectomy in pediatric patients with obstructive sleep apnea syndrome [19]. Furthermore, the performance of adenotonsillectomy improved the sleep-related quality of life and behavioral parameters in children with OSAS [20].  Nevertheless, in some unique patient groups several risk factors may contribute greatly to more repeat tube insertion (TTIs). For instance, the younger age and COME were recognized as the adjusted risk factors for TTIs in children with Down syndrome [22] |
